# A Novel Frailty Score Based on Laboratory Parameters (FIMS Score) for the Management of Older Patients with Severe Aortic Stenosis

**DOI:** 10.3390/jcm12185927

**Published:** 2023-09-12

**Authors:** Augusto Esposito, Ilenia Foffa, Luca Bastiani, Cecilia Vecoli, Antonio Rizza, Simona Storti, Alberto Ranieri De Caterina, Annamaria Mazzone, Sergio Berti

**Affiliations:** 1 Cardiology Unit, Ospedale del Cuore, Fondazione Toscana “G. Monasterio”, 54100 Massa, Italy; rizza@ftgm.it (A.R.); adecaterina@ftgm.it (A.R.D.C.); berti@ftgm.it (S.B.); 2 Institute of Clinical Physiology, National Research Council, Via Aurelia Sud, 54100 Massa, Italy; ilenia.foffa@cnr.it (I.F.); luca.bastiani@cnr.it (L.B.); cecilia.vecoli@cnr.it (C.V.); 3 Fondazione Toscana Gabriele Monasterio, Via Aurelia Sud, 54100 Massa, Italy; simona.storti@ftgm.it (S.S.); mazzone@ftgm.it (A.M.)

**Keywords:** sarcopenia, frailty, outcome, TAVR, aortic stenosis, score

## Abstract

This study aimed to develop a novel score based on common laboratory parameters able to identify frail and sarcopenic patients as well as predict mortality in elderly patients with severe aortic stenosis (AS) for tailored clinical decision-making. A total of 109 patients (83 ± 5 years; females, 68%) with AS underwent a multidisciplinary pre-operative assessment and finalized a “frailty-based management” for the AS interventional treatment. Laboratory parameters of statistically significant differences between sarcopenic and non-sarcopenic individuals were tested in the structural equation model (SEM) to build a Frailty Inflammation Malnutrition and Sarcopenia score (FIMS score). Mortality at 20 months of follow-up was considered an outcome. FIMS score, in particular, the cut-off value ≥ 1.28 was able to identify “frail” and “early frail” patients and predict mortality with a sensitivity of 83.3% and 82.6%, respectively (*p* = 0.001) and was an independent determinant associated with a higher risk of mortality (HR 5.382; *p*-value = 0.002). The FIMS score, easily achievable and usable in clinical practice, was able to identify frail and sarcopenic patients as well as predict their adverse clinical outcomes. This score could provide appropriate guidance during decision-making regarding elderly patients with severe AS.

## 1. Introduction

Frailty is a generic term usually referring to a geriatric syndrome characterized by decreased physiological reserve and increased vulnerability to stressors. On the other hand, sarcopenia is an age-related progressive loss of muscle mass and strength associated with a poor prognosis for several clinical outcomes [1]. Frailty and sarcopenia may exert synergistic adverse effects, resulting in physical and functional impairments.

In recent years, there has been an increasing interest in frailty as a predictor of outcomes after transcatheter aortic valve replacement (TAVR), thus guiding the risk stratification of those patients before the procedure [2]. Several validated clinical frailty scales are used to predict midterm mortality and progressive disability after an aortic valve procedure. Conversely, there is no single diagnostic criterion for the establishment of sarcopenia, although low handgrip strength (HGS) is one of the more commonly used. In the last consensus published by the European Working Group on Sarcopenia in Older People (EWGSOP), it was recommended to measure muscle strength by handgrip using dynamometry and muscle mass mainly by dual-energy X-ray absorptiometry or bioimpedance analysis [3]. However, this condition is still overlooked in clinical practice, and despite a common effort toward the identification of parameters able to predict clinical and surgical outcomes [4], a simple and valid method to identify sarcopenia in clinical settings is needed. In particular, the assessment of frailty and sarcopenia may be a useful prognostic tool to predict perioperative clinical and surgical outcomes and may be an important part of the preoperative decision-making process for surgical strategies.

Innovative multimarker analytical strategies to identify candidate biomarkers involved in pathways that are shared between frailty and sarcopenia may support the development of new tools for a more accurate identification of frailty and sarcopenia and the design of interventions to delay, prevent, or alleviate the associated negative outcomes.

The aim of this paper was to develop and test a novel, repeatable, and simple score based on routine clinical chemistry laboratory parameters that may be useful to identify frailty as well as predict mortality in patients with severe aortic stenosis (AS).

## 2. Methods

### 2.1. Study Population

We evaluated data derived from a pilot clinical project for elderly patients with severe AS (109 elderly patients; median age ± SD, 83.3 ± 5.5; 32% males) to optimize the TAVR pathway. Clinical signs, surgical risk, nutritional markers, cognitive status, disability, comorbidity, physical frailty, and additionally, Fried criteria were used to refer to tailored management of elderly patients with AS among surgical aortic valve replacement (SAVR), transcatheter aortic valve replacement (TAVR), balloon aortic valvuloplasty (BAV), or medical therapy (MT) [5]. The study was approved by the local Ethics Committee (No. 22239). All patients were scheduled for a follow-up of global and cardiovascular mortality at 20 months. In all patients, a venous blood sample was withdrawn, and routine clinical chemistry parameters including hemoglobin (Hb), neutrophils, lymphocytes, monocytes, total cholesterol, uricemia, creatinine, mean platelet volume (MPV), platelet-large cell ratio (P-LCR), C-reactive protein (CRP), hematocrit (HCT), creatine phosphokinase (CPK), troponin I, gamma-glutamyl transferase (GGT), thyroid stimulating hormone (TSH), high-density lipoprotein cholesterol (HDL-C), low-density lipoprotein cholesterol (LDL-C), brain natriuretic peptide (BNP), and albumin were evaluated.

### 2.2. Statistical Analysis

All statistical analyses were completed using Stata/SE 13.1 and SPSS Version 24. Categorical variables were expressed as percentages, and continuous variables were expressed as mean and standard deviation (SD). First, descriptive analyses were conducted to describe the population. For the power of the study, we chose a medium effect size of 0.3. Considering this effect size, a study power (1-β) of 0.90 was expected with an α value of 0.05. Clinical chemistry parameters are statistically different between sarcopenic and non-sarcopenic individuals, diagnosed using handgrip strength (HGS) (cut-off value < 29 kg in men and <17 kg in women) [6], were tested in the structural equation model (SEM) to build a Frailty, Inflammation, Malnutrition, and Sarcopenia score (FIMS score). The t-student’s test for independent samples was performed using a *p*-value ≤ 0.20 to select the clinical chemistry variables to be included in the SEM model [7]. The SEM overall model fit was assessed using two statistical approaches: a Root Mean Square Error of Approximation (RMSEA) (values between 0.05 and 0.08 indicate acceptable fit, and values <0.05 a good fit) and a Standardized Root Mean Square Residual (SRMR) (values <0.05 indicate a good fit) [8]. Following the SEM analysis, the score represents the weighted sums of the values of selected clinical chemistry laboratory parameters, rescaled to a range of 0–10 using the following formula: 10 × (FIMS − min)/(max − min). The result of this equation was adopted as the FIMS score for each individual, a continuous trait that describes the severity of sarcopenia. Furthermore, the FIMS score was tested by the Kruskal–Wallis test with Bonferroni correction to assess the median differences among frailty groups (pre-frail, early frail, and frail) and for types of treatment (SAVR/TAVR, BAV, and MT). The correlations between the score and the MGA indices were evaluated using Pearson’s correlation analyses. Finally, the receiver operating characteristic (ROC) curve was applied to evaluate the predictive utility of the developed score. The area under the curve (AUC), optimal cut-off value, sensitivity, and specificity were calculated. According to the cut-off value of the score, the population was divided into two groups. A multivariable Cox proportional hazards model analysis was used to evaluate the prognostic value of the FIMS score on mortality during the follow-up period.

## 3. Results

### 3.1. Characteristics of the Study Population

In 109 elderly patients with symptomatic severe AS, we identified 27.5% of frail patients and 49.5% of sarcopenic subjects. According to sarcopenia diagnosis based on HGS (Table 1), the two groups, sarcopenic and non-sarcopenic patients, did not differ in age or gender. In the sarcopenic group, we found 55.5% of frail patients, 25.9% were treated with MT, 40.8% underwent BAV, and 33.3% of sarcopenic patients underwent TAVR treatment. Among sarcopenic patients, nobody underwent SAVR.

Clinical chemistry analyses showed that neutrophils, BNP, creatinine, CPK, and Troponin I showed statistically significant differences between sarcopenic and non-sarcopenic individuals (Table 2), whereas Hb, CRP, HDL-C, and albumin were different with a *p*-value ≤ 0.20.

### 3.2. FIMS Score and Correlation

**Figure 1:**
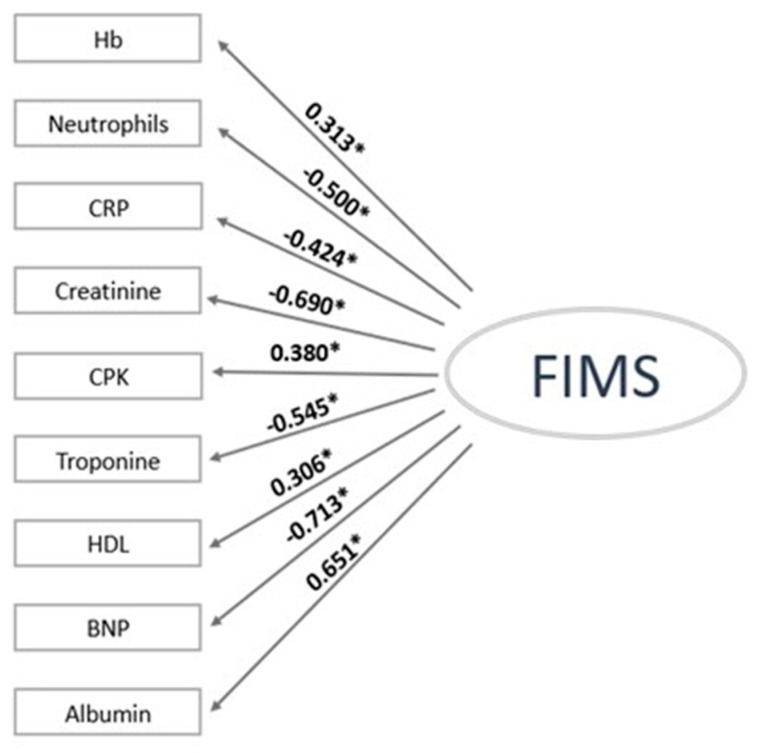
Standardized factor loading values of the single factor model of Sarcopenia. The FIMS score, following the structural equation model analysis, represents the weighted sums of the values of selected clinical chemistry laboratory parameters. The score has been rescaled to a range of 0–10 using the following formula: 10 × (FIMS − min)/(max − min). The goodness-of-fit indices are as follows: a standardized root mean square residual of 0.052 and a root mean square error of approximation of 0.072. * Statistical level of significance *p*-value < 0.05.

Figure 1 shows the values of standard laboratory parameters selected to build the FIMS (Fragility Inflammation Malnutrition Sarcopenia) score.

The magnitude of each factor loading value was >0.3, which indicated the importance of the corresponding index to the sarcopenia. BNP, creatinine, and albumin were the most weighted variables, with factor loading values of −0.713, −0.690, and 0.651, respectively. The other variables with good factor loading were Troponin I (−0.545), Neutrophils (−0.500), and CRP, with item-factor correlations of −0.424. The lowest values were observed for the CPK, Hb, and HDL, which had factor loadings of 0.380, 0.313, and 0.306, respectively. The goodness of fit (i.e., SMSR and RMSEA) of the FIMS was acceptable because the indices were <0.10 (SMSR 0.052; RMSEA 0.072) (Figure 1). Accordingly, the FIMS score for each individual can be considered a continuous trait describing the severity of the sarcopenia.

### 3.3. FIMS Score, Frailty, and Multidimensional Geriatric Assessment

**Figure 2:**
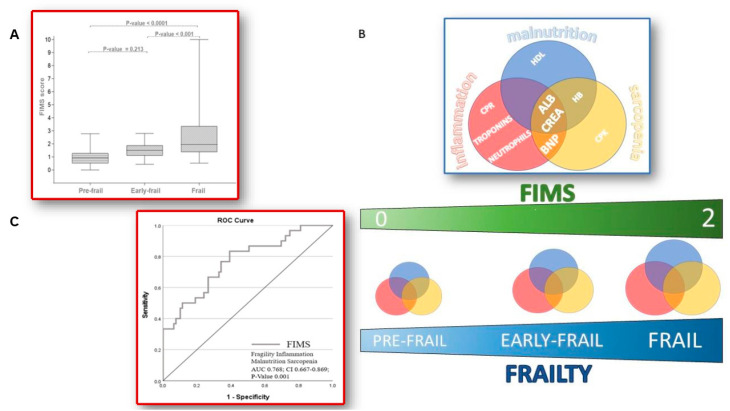
FIMS score and frailty (**A**) The median FIMS score between classes of frailty (**B**) A graphic representation of the median value of the FIMS score between classes of fragility and the relationship between laboratory parameters and malnutrition, inflammation, and sarcopenia in frailty patients with severe AS (**C**) ROC curve of FIMS score diagnostic accuracy for frailty.

The median and IQR of FIMS scores were calculated for “pre-frail” (0.93; 0.51–1.28), “early-frail” (1.51; 1.1–1.90), and “frail” (1.96; 1.38–3.35) patients, previously categorized by Fried’s criteria. By comparing the median FIMS score, “frail” patients were statistically significantly different, concerning “pre-frail” (*p* < 0.0001) and “early-frail” (*p* = 0.001) patients. Conversely, no significant difference was observed between the “pre-frail” and “early-frail” groups (*p* = 0.213) (Figure 2A,B).

The predictive utility of the developed FIMS score to assess frailty was evaluated using ROC curve analysis. The study population was divided into two groups: pre- and early-frail patients and frail patients. The area under the ROC curve (AUC) value was 0.768 (CI 0.667–0.869; *p* = 0.001). The cut-off point was ≥1.28, which showed a sensitivity of 83.3% and a specificity of 60.0% to predict frailty (Figure 2C).

**Figure 3:**
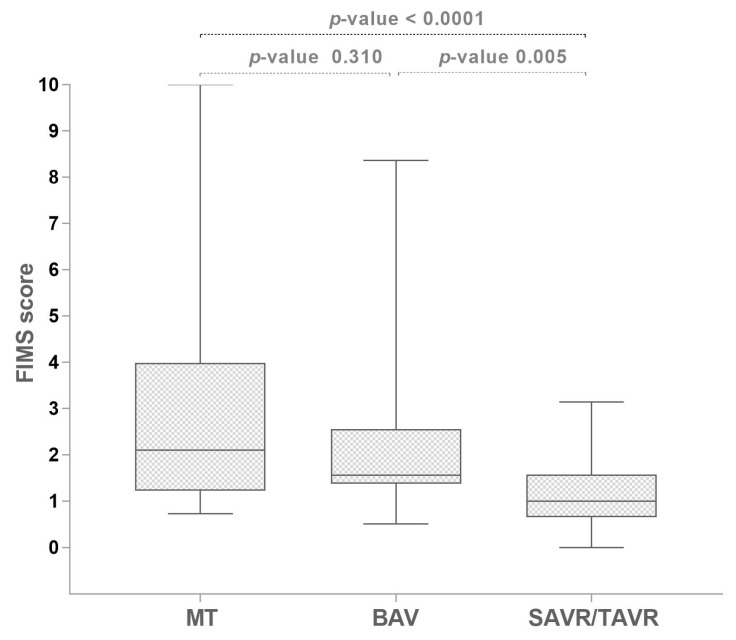
Graphic representation of FIMS factor scores between types of treatment.

Patients under MT or undergoing BAV according to their frailty condition (defined by Fried score) showed a FIMS median value statistically significantly higher than patients who underwent SAVR/TAVR (Figure 3).

Moreover, the FIMS score showed a statistically significant correlation with standardized MGA indices. In particular, the score was negatively related to basic activities of daily living (BADL), instrumental activities of daily living (IADL), and Mini Nutritional Assessment (MNA) (*p* < 0.05) and positively related to the Charlson Comorbidity Index (*p* < 0.05) while it did not correlate with the mini-mental state examination for cognitive function evaluation (MMSE) (Table 3).

In addition, the FIMS score was higher in patients with NYHA III-IV than in class II (*p* = 0.002).

### 3.4. FIMS Score as a Predictor of Mortality

**Figure 4:**
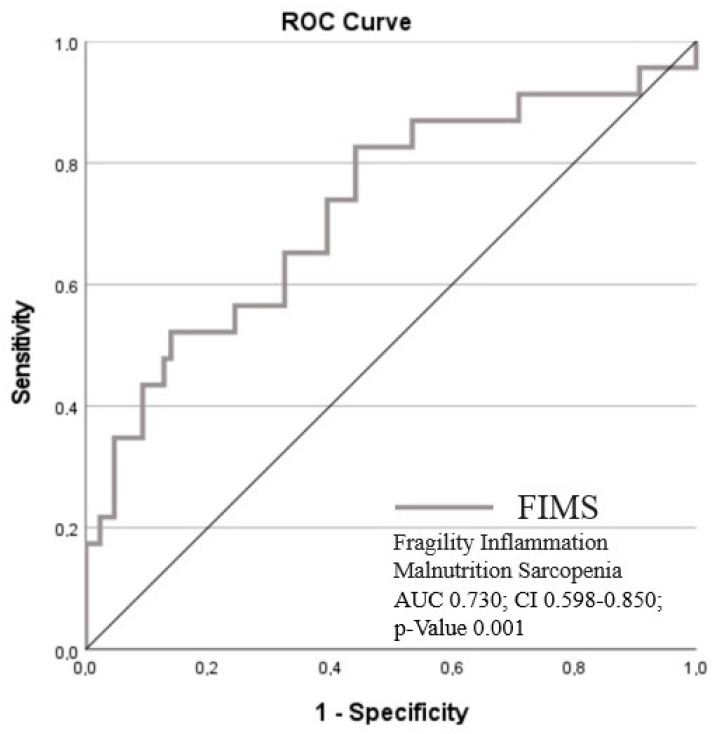
ROC curve of FIMS score for adverse outcome prediction.

During the 20 months of follow-up, 23 all-cause deaths were registered. The median of FIMS scores was lower in surviving patients (2.10, 1.33–2.90 vs. 1.22, 0.74–1.77, *p*-value = 0.001). The area under the ROC curve (AUC) values for 20-month survival were calculated to be 0.730 (CI 0.598–0.85; *p* = 0.001). The cut-off point was ≥1.28, which showed a sensitivity of 82.6% and a specificity of 57.8% to predict the adverse outcome (Figure 4).

The multivariate Cox regression model (adjusted for age and sex) showed that the cut-off FIMS score value ≥ 1.28 was an independent determinant associated with a higher risk of mortality during the follow-up period (HR 5.382; CI 95% 1.810–15.997; *p*-value 0.002).

## 4. Discussion

As the population ages, recent years have seen increased interest in frailty and sarcopenia in health policy and research. Both frailty and sarcopenia are considered potentially reversible conditions [9], and therefore, their early detection is of the utmost importance to prevent subsequent functional decline and disability as well as predict mortality risk. However, it is not always feasible to perform an accurate assessment in clinical settings. Frailty indexes usually include some physical tests and questionnaires, which are time-consuming, while sarcopenia diagnosis requires expensive tests such as dual-energy X-ray absorptiometry (DXA). Therefore, clinicians require valid, easy-to-use tools with automatic data processing and real-time results without complicated interpretation or instrumentation. A clinically affordable diagnostic tool for detecting frailty and sarcopenia syndromes might have implications for prognosis, care optimization, and planning interventions for community-dwelling older adults. In this study, we identified a novel score based on standard laboratory parameters able to identify “Frail” patients according to frailty-based management considering the Fried score and MGA as previously described by Mazzone et al. [5] and to predict mortality in elderly patients with symptomatic severe AS. In particular, we showed that the FIMS score value was significantly higher in patients who, in frailty-based tailored management, underwent MT or BAV. So, in a larger study population, this should be confirmed as helpful in therapeutic decision-making for elderly patients with aortic stenosis. Moreover, we showed that the cut-off FIMS score value ≥ 1.28, after adjustments for potential confounders, remained an independent predictor of mortality and correlated with other traditional multidimensional geriatric assessment instruments. So, this simple tool, which correlates with the traditional score, could be applied in a pre-operative assessment and finalized for “frailty-based management” of elderly patients to perform a personalized approach.

As regards the physiopathological aspects, the FIMS score supports the role of malnutrition, inflammation, sarcopenia, and physical frailty in adverse outcomes in elderly patients with severe aortic stenosis. Physical frailty and sarcopenia are related syndromes, sharing several features such as lower lean mass and reduced physical function [10]. Malnutrition seems to play a key role in the pathogenesis of both of these conditions and vice versa [11], demonstrating the complex and synergistic relationship by which one condition is accelerated by the other.

Therefore, in recent years, the assessments of frailty, sarcopenia, and malnutrition have become a growing field of interest given the association between these clinical states and adverse outcomes, and several studies have tried to describe this relationship [12].

Frailty development is partly dependent on multiple factors such as low levels of nutrients and high levels of oxidative stress and inflammation, potentially leading to a muscle-catabolic state. Moreover, epidemiological and pathophysiological evidence showed that sarcopenia is likely the key element that links physical frailty and malnutrition [13].

The laboratory’s biomarkers used to create the FIMS score show a shared core between malnutrition, inflammation, sarcopenia, and frailty—aspects that are apparently separated but probably strictly interconnected with each other. Albumin, creatinine, and BNP were the most impactful variables among the biomarkers used to create the FIMS score. Serum albumin is a marker of systemic inflammation. Indeed, in a state of chronic inflammation, the liver responds by producing several acute-phase molecules while the synthesis of a number of other proteins, including albumin, is depressed. That a decreased protein synthesis may serve to save amino acids for producing “positive” acute-phase proteins more efficiently. This might explain the negative association of albumin with frailty and sarcopenia. Low levels of albumin are also a surrogate marker of nutritional status, and hypoalbuminemia represents a risk factor for perioperative complications [14,15]. Malnutrition, routinely indicated by low serum albumin, often coexists with sarcopenia and may accelerate the muscular degeneration process. In particular, serum albumin levels are often reduced in patients with AS [16] and are associated with an increased risk of mortality after TAVR [17]. High levels of serum creatinine are typically present in the elderly population as a result of increased production during skeletal muscle metabolism and reflect muscle weakness [18,19].

Heart failure (HF) based on a valvular cause, such as severe AS, is often accompanied by cardiac cachexia and, as a consequence, sarcopenia [20]. The BNP is released by myocardial cells in response to increased parietal stress under conditions of volume or intramyocardial pressure overload. BNP has a peripheral vasodilator effect that contributes to reducing this intramyocardial overload. It has been shown that patients with sarcopenia have higher BNP levels compared with those without sarcopenia [21]. In line with this evidence, elevated baseline levels of NT-pro BNP have been associated with mortality after TAVR [22]. Accordingly, in our elderly population of patients with AS, significantly higher serum levels of BNP were found in sarcopenic patients.

The other variables with good factor loading in the FIMS score were CRP, neutrophils, and Troponin I. Low-grade chronic inflammation, displayed by a high serum level of CRP, plays a key role in the pathophysiology of frailty and loss of physical performance, which are closely associated with sarcopenia. As well described by Shokri-Mashhadi et al., the decreased muscle strength was independently associated with high CRP serum levels [23]. Moreover, our data also confirm the association between neutrophils and frailty measures, as already reported by Collerton et al. [24].

In our study population, we found significantly higher levels of Troponin I in sarcopenic patients with AS compared with non-sarcopenic ones.

Several studies demonstrated that the long-term outcome of TAVR critically depends on the myocardial damage that evolved before intervention [25] and that the chronic elevation of the serum concentration of cardiac troponins, without symptoms of ischemia, is the clinical hallmark of such myocardial injury [26]. In addition, high levels of cardiac troponin are associated with increased mortality and cardiovascular disease morbidity in the general population [27] and patient population [28]. In particular, baseline high-sensitivity cardiac Troponin T before TAVR is strongly associated with the composite risk of all-cause death and rehospitalization during long-term follow-up [25]. Lastly, despite the lowest value of magnitude, our data demonstrated an independent association between Hb, HDL, CPK, and sarcopenia.

According to recently published studies by Tseng et al. [29] and Bani Hassan et al. [30], our data demonstrated an independent association between Hb levels and sarcopenia. Moreover, Hecht et al. showed Hb significantly decreased in sarcopenic TAVR patients compared to non-sarcopenic patients [31]. Low levels of Hb might suggest reduced hemopoiesis as a pathway to sarcopenia. Anemia causes reduced tissue oxygenation that may have a crucial impact on tissues highly dependent on oxidative metabolism, such as the muscle, with consequent fatigue, weakness, and increased functional impairment.

According to our results, Chung et al. showed that the mean values of HDL-cholesterol were significantly lower in subjects with sarcopenia than in subjects without sarcopenia for both men and women [32] and Formiga et al. reported that lower HDL-C values were associated with poor MNA scores and therefore with malnutrition [33]. Interestingly, Yelgeç et al. [34] investigated the effect of serum cholesterol levels on the prognosis of TAVR patients. In line with our results, lower levels of HDL cholesterol were associated with a poor prognosis after TAVR, suggesting that a measurement of serum lipid levels might improve the preoperative risk assessment of potential TAVR candidates. Circulating creatine phosphokinase (CPK) is clinically used as an indicator of muscle damage and a diagnostic of such conditions as myocardial infarction and severe muscle breakdown. Low levels of CPK reflect an aged skeletal muscle, as confirmed in a proteomic study in which selected candidate proteins confirmed the effect of aging on the skeletal muscle proteome [35]. Moreover, according to our data, CPK was also used as a factor in another risk model, developed by Maeda et al., for a prognostic prediction after TAVR [36].

Thus, our results confirm the role of malnutrition and inflammation in sarcopenia and frailty in patients with severe and symptomatic AS, underlining the interplay between frailty, malnutrition, sarcopenia, and TAVR outcomes. In addition, since it is well known that early detection of these syndromes might potentially increase the chances of reversibility, it could be recommended to manage/treat malnutrition, frailty, and/or sarcopenia, given the interplay of these three conditions, to improve the TAVR outcome.

## 5. Limitations

This study was limited by its retrospective nature and was performed at a single center with a relatively small number of patients. However, despite these limitations, using a well-characterized population with a multidisciplinary approach, our study has some important conclusions. Moreover, as reported in the Statistical Analysis Section, a study power of 0.90 was expected (with a medium effect size of 0.3 and a *p*-value of 0.05).

## 6. Conclusions

The FIMS score can be used to define frail and sarcopenic patients and to predict adverse outcomes in an elderly population with aortic stenosis for a personalized approach, suggesting that it could potentially guide the decision-making process of treatment strategies and that it could be adopted in routine care of older people. Future large-scale multicenter prospective studies in different aging populations will confirm the utility of the FIMS score to support therapeutic decision-making. This simple tool could be easily used in clinical settings to identify frail patients with a higher risk of adverse outcomes in other aging populations.

## Figures and Tables

**Table 1:** Characteristics of the study population between sarcopenic and non-sarcopenic patients.

			Sarcopenia		
		Total (N = 109)	Yes (N = 54)	No (N = 55)	*p*-Value
Age	Mean; Sd	83.33 (5.5)	83.8 (6.3)	82.9 (4.6)	0.420
Gender	Female	67.9%	72.2%	63.6%	0.337
	Male	32.1%	27.8%	36.4%	
Fried frailty phenotype	Pre-frail	41.3%	13.0%	69.1%	0.0001
	Early-frail	31.2%	31.5%	30.9%	
	Frail	27.5%	55.5%	0.0%	
Treatment classes	Surgical aortic valve replacement (SAVR)	7.3%	0.0%	14.5%	0.0001
	Medical therapy (MT)	13.8%	25.9%	1.8%	
	Balloon aortic valvuloplasty (BAV)	24.8%	40.8%	9.1%	
	Valve replacement (TAVR)	54.1%	33.3%	74.6%	

**Table 2:** Laboratory parameters differ between sarcopenic and non-sarcopenic patients.

	Sarcopenia				*p*-Value
	Yes (N = 54)		No (N = 55)		
	Mean	Sd	Mean	Sd	
**Haemoglobin (Hb), g/dL**	12.12	1.8	12.64	1.5	0.112
**Neutrophils, n/µL**	5588.52	2202.2	4560.00	1614.4	0.006
Lymphocytes, n/µL	1712.26	1005.7	1871.38	851.3	0.374
Monocytes, n/µL	662.22	211.6	626.36	213.0	0.406
**C-reactive protein (CRP), mg/dL**	0.95	1.9	0.54	0.9	0.161
Total cholesterol, mg/dL	182.50	28.9	187.74	41.7	0.457
Uricemia, mg/dL	6.26	2.1	5.90	1.7	0.380
**Creatinine, mg/dL**	1.62	1.6	1.08	0.3	0.015
Mean Platelet Volume (MPV), fl	10.83	0.9	10.69	0.8	0.401
Haematocrit (HCT), %	37.36	5.2	38.45	4.2	0.233
**Creatine phosphokinase (CPK), IU/L**	57.34	27.9	80.80	40.7	0.001
**Troponin I, µg/L**	0.04	0.0	0.02	0.0	0.027
Gamma-Glutamyl Transferase (GGT), IU/L	29.75	37.4	33.19	35.7	0.630
Thyroid-Stimulating Hormone (TSH), mU/L	1.78	1.1	3.17	9.8	0.306
**High-Density Lipoprotein cholesterol (HDL), mg/dL**	54.06	17.9	58.13	13.2	0.190
Low-Density Lipoprotein Cholesterol (LDL), mg/dL	107.73	26.6	105.94	33.7	0.768
**B-type natriuretic pepetide (BNP), mg/L**	746.17	1227.7	367.37	497.8	0.048
**Albumin, g/dL**	3.91	0.5	4.11	0.4	0.020

**Table 3:** Correlations between FIMS score and Multidimensional Geriatric Assessment indices.

Variables		FIMS Score
	r	*p*-Value
Instrumental Activities of Daily Living (IADL)	−0.33	<0.0001
Basic Activities of Daily Living (BADL)	−0.279	0.003
Mini Nutritional Assessment (MNA)	−0.316	0.001
Mini Mental State Examination (MMSE)	−0.47	0.6
Charlson Comorbidity index	0.371	<0.0001

## Data Availability

The data underlying this article will be shared on reasonable request to the corresponding authors.
